# Trends in Smart Helmets With Multimodal Sensing for Health and Safety: Scoping Review

**DOI:** 10.2196/40797

**Published:** 2022-11-15

**Authors:** Peter Lee, Heepyung Kim, M Sami Zitouni, Ahsan Khandoker, Herbert F Jelinek, Leontios Hadjileontiadis, Uichin Lee, Yong Jeong

**Affiliations:** 1 KAIST Institute for Health Science and Technology Korea Advanced Institute of Science and Technology Daejeon Republic of Korea; 2 College of Engineering and IT University of Dubai Dubai United Arab Emirates; 3 Department of Biomedical Engineering Khalifa University of Science and Technology Abu Dhabi United Arab Emirates; 4 Healthcare Engineering Innovation Center Khalifa University of Science and Technology Abu Dhabi United Arab Emirates; 5 Department of Electrical and Computer Engineering Aristotle University of Thessaloniki Thessaloniki Greece; 6 School of Computing Korea Advanced Institute of Science and Technology Daejeon Republic of Korea; 7 Department of Bio and Brain Engineering Korea Advanced Institute of Science and Technology Daejeon Republic of Korea

**Keywords:** Internet of Things, IoT, sensor technology, smart helmet, smart sensor, wearable device, mobile phone

## Abstract

**Background:**

As a form of the Internet of Things (IoT)–gateways, a smart helmet is one of the core devices that offers distinct functionalities. The development of smart helmets connected to IoT infrastructure helps promote connected health and safety in various fields. In this regard, we present a comprehensive analysis of smart helmet technology and its main characteristics and applications for health and safety.

**Objective:**

This paper reviews the trends in smart helmet technology and provides an overview of the current and future potential deployments of such technology, the development of smart helmets for continuous monitoring of the health status of users, and the surrounding environmental conditions. The research questions were as follows: What are the main purposes and domains of smart helmets for health and safety? How have researchers realized key features and with what types of sensors?

**Methods:**

We selected studies cited in electronic databases such as Google Scholar, Web of Science, ScienceDirect, and EBSCO on smart helmets through a keyword search from January 2010 to December 2021. In total, 1268 papers were identified (Web of Science: 87/1268, 6.86%; EBSCO: 149/1268, 11.75%; ScienceDirect: 248/1268, 19.55%; and Google Scholar: 784/1268, 61.82%), and the number of final studies included after PRISMA (Preferred Reporting Items for Systematic Reviews and Meta-Analyses) study selection was 57. We also performed a self-assessment of the reviewed articles to determine the quality of the paper. The scoring was based on five criteria: test environment, prototype quality, feasibility test, sensor calibration, and versatility.

**Results:**

Smart helmet research has been considered in industry, sports, first responder, and health tracking scenarios for health and safety purposes. Among 57 studies, most studies with prototype development were industrial applications (18/57, 32%), and the 2 most frequent studies including simulation were industry (23/57, 40%) and sports (23/57, 40%) applications. From our assessment-scoring result, studies tended to focus on sensor calibration results (2.3 out of 3), while the lowest part was a feasibility test (1.6 out of 3). Further classification of the purpose of smart helmets yielded 4 major categories, including activity, physiological and environmental (hazard) risk sensing, as well as risk event alerting.

**Conclusions:**

A summary of existing smart helmet systems is presented with a review of the sensor features used in the prototyping demonstrations. Overall, we aimed to explore new possibilities by examining the latest research, sensor technologies, and application platform perspectives for smart helmets as promising wearable devices. The barriers to users, challenges in the development of smart helmets, and future opportunities for health and safety applications are also discussed. In conclusion, this paper presents the current status of smart helmet technology, main issues, and prospects for future smart helmet with the objective of making the smart helmet concept a reality.

## Introduction

### Background

An Internet of Things (IoT)–smart helmet can be defined as a helmet integrated with electronic sensing, alerting, and communication devices and used not only as protective headgear but also to provide intelligent services for enhancing user capabilities or minimizing the risk of injuries and fatalities. This technology can benefit users through activity [1], physiological [2], and environment [3] monitoring or location sharing [4]. With advances in computing power and microelectronics, health and safety can be further promoted with real-time physiological measurement capabilities and data processing that can provide actionable and important information about an individual and their surrounding environment [5-9]. Specifically, smart helmets, as personal protective equipment, can be used to reduce injuries, ensure safety, especially in hazardous occupations, and make the return of injured people fast and easy. However, because of the recent development of smart helmets, previous publications lack an in-depth review of existing studies on the diversity of smart helmets and possible future innovations for health and safety purposes. Similar to wearable sensors, smart helmets are being increasingly used to address health and safety concerns [10]. Currently, available commercial systems and research are mainly focused on motorcycle helmets and applications for defense personnel, where wearing a smart helmet is mandatory or recommended.

Previous studies have indicated the potential applications of smart helmets in diverse scenarios, such as industry, first responder applications, and health tracking [11]. For instance, the construction industry has adopted the use of smart helmets for health and safety management by monitoring workplace surroundings or the physiological signals of workers [12]. As construction workers are prone to falls and objects falling, most studies considered inertial measurement unit (IMU) sensors to detect accidents [13-16]. A study by Seo et al [17] and Pirkl et al [18] used ultrasonic sensors to quantify space dimension by measuring wave bounding and floor safety by measuring the density of the walls. Other industrial applications include a DAQRI smart helmet that used augmented, mixed reality for user enhancements like object recognition, resource management, and thermal vision [19]. For a motorcyclist, a smart helmet can send a message to the nearest hospital if the rider is involved in an accident [20,21]. Moreover, an alcohol sensor can measure the alcohol level of the rider and lock the ignition system if the level is above a certain threshold [22]. For first responders, a smart helmet can provide a thermal scan of an individual to check for COVID-19 symptoms [23] or injuries such as broken bones [24] or bleeding [25]. An IoT smart helmet can also track the status of the response crew in real time and report back to a central control center [23]. With the development of the Internet of Battlefield Things, smart helmets are becoming more prominent in the military [26]. Furthermore, smart helmets can be used as health trackers to acquire physiological, behavioral, and contextual data for the diagnosis, treatment, and management of chronic diseases and negate the risk of injury such as falls in the older adults [27].

There are several studies on smart helmet technology that focus on specific fields of application. For instance, Fernández-Caramés et al [28] introduced a smart helmet as part of smart clothing and combined it with smart glasses for augmented reality. Similarly, Campero-Jurado et al [29] described the possible integration of artificial intelligence technology with smart helmets to improve the safety of workers. Mardonova et al [30] described the use of wearable sensor technology in the mining industry and other applications to enhance the safety of mining operations and improve wellness among workers. Similarly, Shi et al [26] reviewed wearable device applications for the military and proposed a framework based on body sensor networks.

### Objectives

This review presents a comprehensive analysis of smart helmet technology and its main characteristics and applications for health and safety and provides a presentation of the most relevant challenges to its implementation. Thus, our overarching goal is to clarify the main purpose and domain of smart helmet technologies to improve health and safety through sensing, inference, and actuation. This is achieved by addressing the following questions:

1. What are the main purposes and domains of smart helmets for health and safety?
2. How have researchers realized key features (inference and actuation) and with what types of sensors (sensing)?

The remainder of this paper is organized as follows. After selecting studies that contain the required keywords, we assessed smart helmet articles to ensure the quality of the prototype. We then classified and summarized the studies based on their domain, purpose, and sensor use. Finally, we have discussed the findings and reviewed the trends in smart helmet technology, identifying the main current technical limitations and outlining the primary challenges on a broader scale.

## Methods

This review was performed according to the PRISMA (Preferred Reporting Items for Systematic Reviews and Meta-Analyses) guidelines [31]. Accordingly, strict eligibility criteria were applied to identify journal articles and reviews addressing the collection of sensor-based smart helmet information and to investigate key features of smart helmets with built-in sensor applications.

### Study Selection Criteria

The search for appropriate studies was performed using the following electronic databases: Web of Science, ScienceDirect, and Google Scholar. The following combination of search terms was used: “Smart helmet” AND “sensor.” Duplicated studies were removed before starting the selection. An eligibility check was performed on the title, keywords, and abstract of each study. Full-text copies of all potentially relevant papers and papers with insufficient information in the abstract to determine eligibility were obtained. The inclusion and exclusion criteria were as follows:

• Types of methods: studies reporting smart sensing using custom sensors such as infrared (IR), ultrasound, piezoelectric, temperature, and GPS sensors were included. Studies describing internet-based interventions, conceptual technologies, and collision testing for helmet design without a sensor-based component were excluded.

• Types of outcomes: studies reporting results associating prototype and sensor-based data were included. Papers providing descriptions of smart helmets but no experimental outcomes were excluded.

• Language and time frame: English-language full-text articles were included in the review. Considering the trend of technology evolution, only papers published between January 2010 and December 2021 were included.

A study selection, in accordance with the eligibility criteria, was performed independently by 2 of the authors: one with a clinical background and one with a technological background. There were no cases of disagreements between the 2 authors.

### Content Analysis and Study Quality Assessment

The extracted information consisted of the following: (1) field, (2) smart helmet purpose, (3) sensors used, and (4) validation. We also performed a self-assessment of quality on reviewed articles based on 5 criteria; that is, test environment, prototype quality, feasibility test, sensor calibration, and versatility of the smart helmet (Textbox 1). Each criterion was scored from 1 to 3, and the sum of scores ranged from 5 to 15, as prior studies used this kind of scoring to provide an overview of the quality of the papers reviewed [32-35]. This idea of *assessment scores* originated from the work of Suri et al [32], AtheroPoint’s artificial intelligence–based Bias—AP(ai)Bias—for detecting a risk of bias in the study selection process. By scoring studies with each attribute, we tried to show the overall trends in smart helmet research. Moreover, the projection of the studies to a common assessment basis provides a unified form for revealing the characteristics that make them advantageous or disadvantageous and can translate these characteristics to interpretable implementations in real-life settings.

**Criteria for quality assessment of reviewed articles.**

**Test environment**

- Daily setting (score 3)
- Controlled setting (score 2)
- Laboratory setting (score 1)

**Prototype quality**

- Complete prototype (score 3)
- Preliminary prototype (score 2)
- Conceptual prototype (score 1)

**Feasibility test**

- Evaluation with cross-validation (score 3)
- Accuracy measurement (score 2)
- Only present sensor test data (score 1)

**Sensor calibration and testing**

- Real test data (score 3)
- Sensor test Data (score 2)
- Only sensor specification data (score 1)

**Versatility**

- Multiple domains with multiple applications (score 3)
- Single domain with multiple applications (score 2)
- Single domain with specific use (score 1)

## Results

### Overview

**Figure 1:**
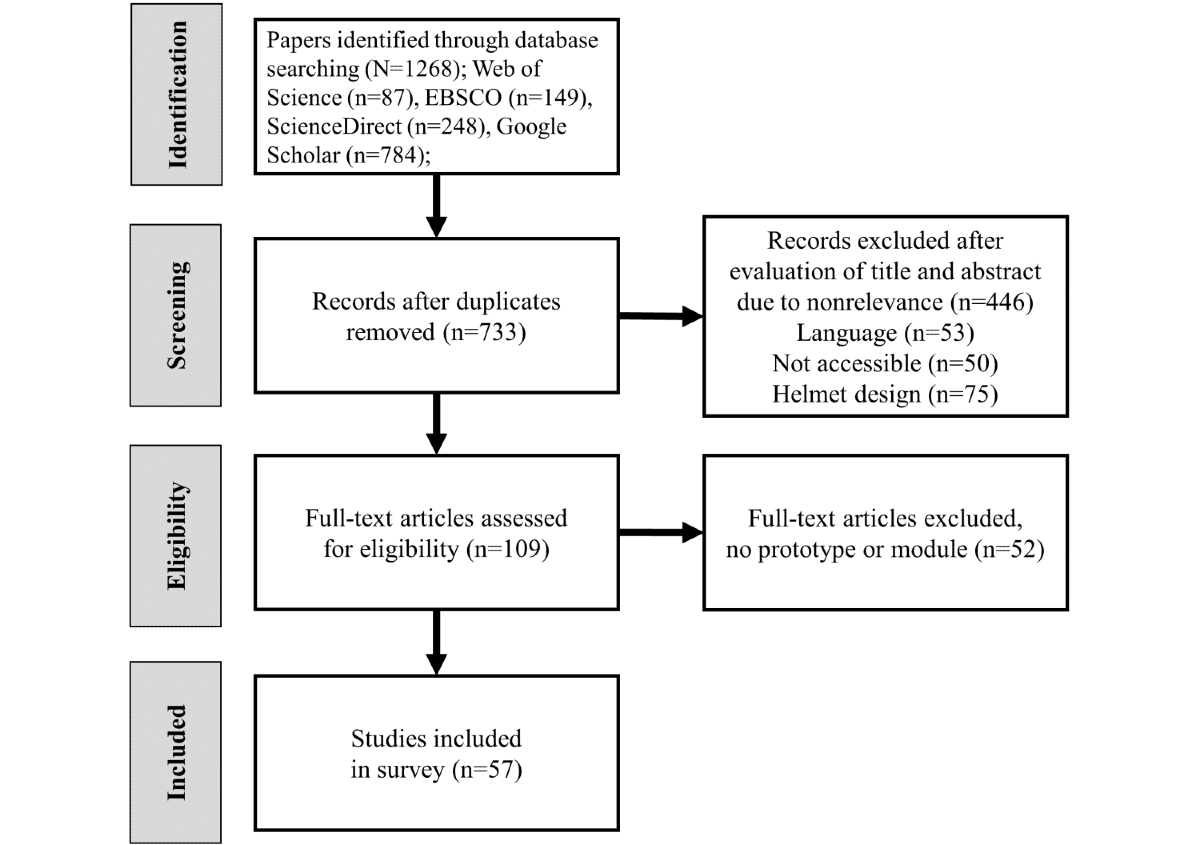
Study selection procedure in flowchart.

As summarized in the PRISMA flowchart in Figure 1, a total of 87 (6.86%) records were obtained from Web of Science, 149 (11.75%) from EBSCO, 248 (19.55%) from ScienceDirect, and 784 (61.83%) from Google Scholar leading to a total of 1268 journal papers. Across the 4 databases, 535 (42.19%) duplicates were identified and removed. A total of 624 additional records were excluded mainly because they reported on other technologies or other fields or both. Some of the papers were written in languages other than English (n=53, 9.9%), not accessible (n=50, 9.3%), or dealt with the design of the helmet (n=75, 14.0%). An additional 52 papers were excluded because they did not report on suitable smart helmets or did not report on sensors. This elimination resulted in 57 full-text papers remaining to be considered.

**Figure 2:**
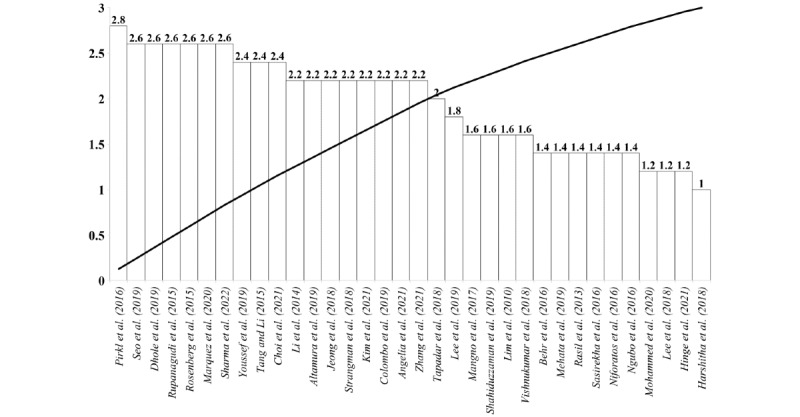
Pareto analysis on smart helmet prototypes.

**Figure 3:**
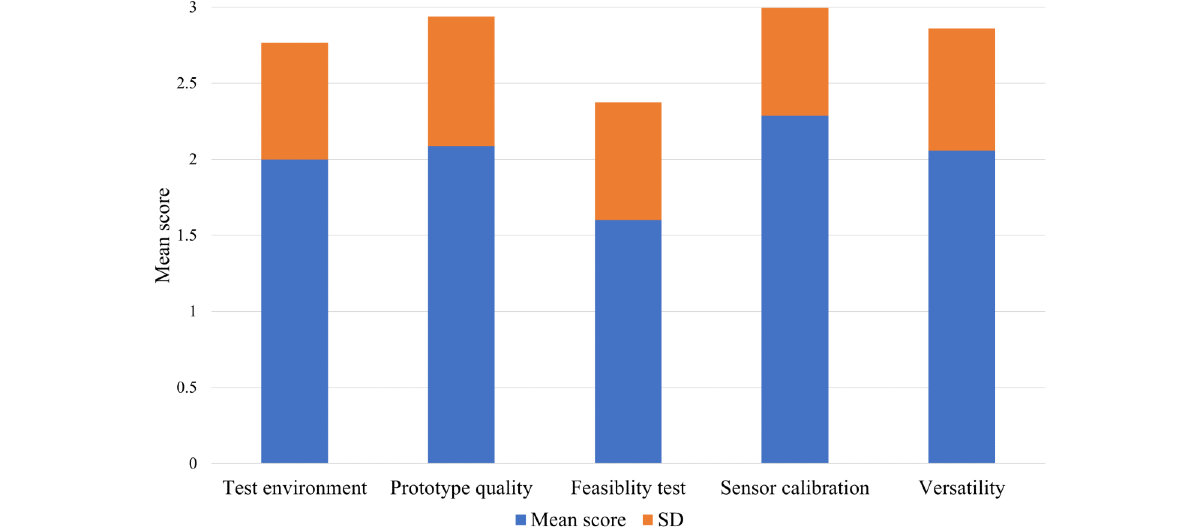
Assessment area score distribution.

To assess the quality of the targeted studies, we ranked the smart helmet prototypes based on the abovementioned scoring criteria. A total of 34 studies provided an actual prototype of the smart helmet and are included in the analysis as illustrated in Figure 2. The raw cutoff mean score was 2.0 with SD 0.5, where higher-than-threshold studies provide a practical and validated smart helmet prototype. The assessment criteria distribution showed that most of the articles focused on sensor calibration while the feasibility test of the prototype was the least scored as presented in Figure 3.

### Classification of Smart Helmets

**Figure 4:**
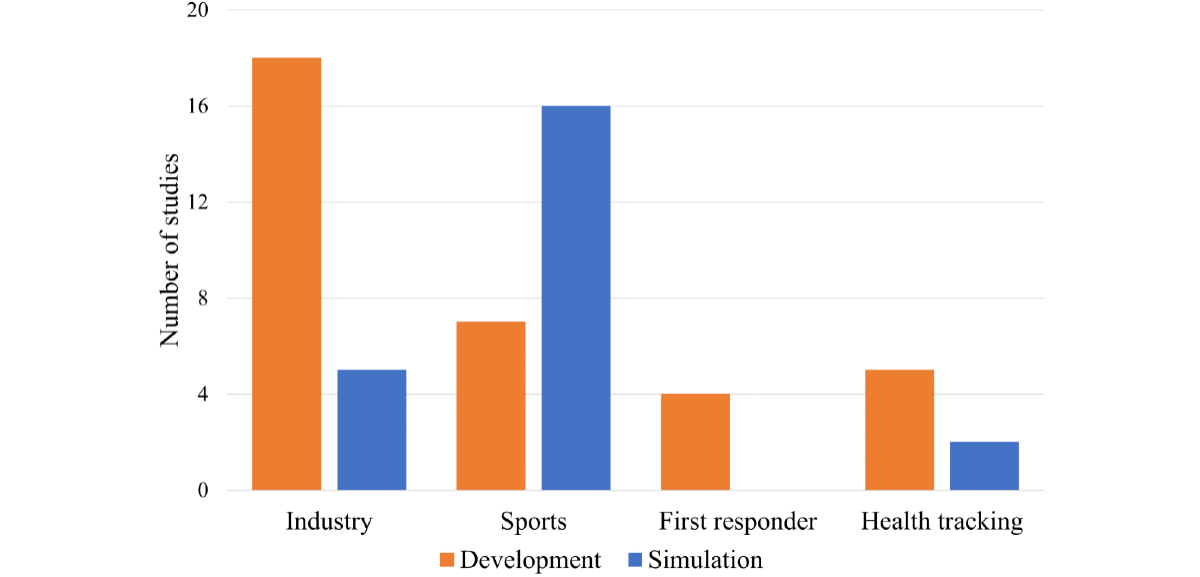
Distribution of articles.

**Figure 5:**
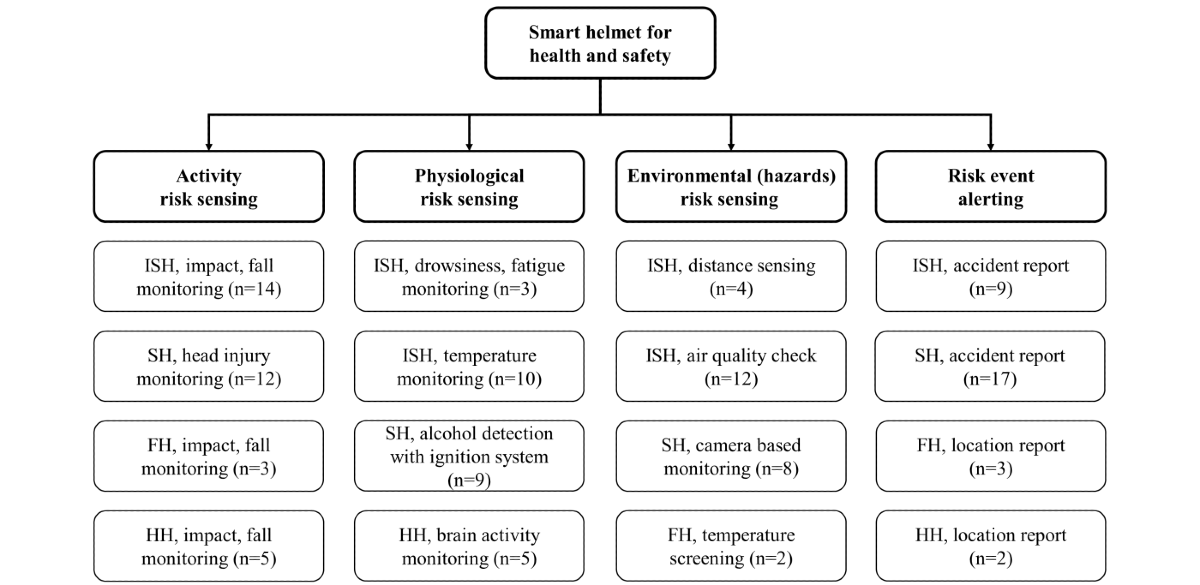
Classification of smart helmets. FH: First responder helmet; HH: health tracking helmet; ISH: industrial safety helmet; SH: sports helmet.

Smart helmets can be classified based on their functions, purposes, and fields of use. Previous studies on smart helmets have mainly been focused on sports, industrial safety, assisting first responders, and health tracking. Figure 4 shows the distribution for the 57 selected articles. The numbers of articles that presented smart helmet prototypes for industry, sport, first responder, and health tracking applications were 18 (53%) [13-18,36-47], 7 (21%) [48-54], 4 (12%) [55-58], and 5 (15%) [59-63], respectively. The numbers of articles that included module simulations instead of prototypes for industry, sport, and health tracking applications were 5 (22%) [64-68], 16 (70%) [69-84], and 2 (9%) [85,86], respectively. Figure 5 provides a further classification of smart helmets based on their purposes for health and safety promotion: activity risk sensing (ARS), physiological risk sensing (PRS), environmental risk sensing (ERS), and risk event alerting (REA).

#### Activity Risk Sensing

As a means of safety, these helmets monitor head motion or impact. Individuals may fall unconscious because of hazardous events and be left without assistance, but sensing via a smart helmet could help mitigate this risk. The sensors used include accelerometers [17,40,49,51,58,60,61,63], gyroscopes [13,60-62], or IMUs [13-16,18,37,44,46,55,57,59]. These sensors can measure 3- to 6-axis bodily motions (eg, acceleration and orientation) and monitor any anomalous events such as sudden spikes of the signal (might indicate fall or hit from a falling object) or become quiet (might indicate no movement or unconscious after an accident) [36,40,55]. The output from these sensors is often integrated with actuation, such as REA systems to enable rapid responses.

#### Physiological Risk Sensing

With the increasing demand for health monitoring, biosensors are incorporated into helmets to allow individuals, health care providers, or sports coaches to track individual, community, or team health status. For instance, industrial workers or firefighters are at risk of fatigue, overheating, and exposure to hazardous gasses where continuous monitoring can prevent such incidents. There have been attempts to quantify worker fatigue using electroencephalography (EEG) signals [13,34], and to use temperature monitoring to manage overheating [14,43,47]. These helmets have integrated sensors for monitoring body temperature [14,15,39,40,42,43,47,52,54,57-60], heart rate [14,40,42,43,57,58], blood pressure [39,47], electrocardiogram (ECG) [59,60,63], and EEG [13,37,59,60,63] signals.

#### Environmental (Hazards) Risk Sensing

In addition to the benefits of monitoring the physiological state of an individual, it is also meaningful to monitor the surrounding environment to minimize risk before an incident happens. For coal-mining workers, exposure to hazardous gasses needs to be avoided and gas sensors incorporated into smart helmets for mining operations can be used to notify users of such gasses [15,16,38,39,42,45]. For health care workers, an IR camera integrated with a head-mounted display can provide thermal scanning to check for COVID-19 or other viral and bacterial infections [14,23,87,88]. A camera mounted on the rear of a smart helmet can scan and warn drivers of motor vehicles or objects approaching from behind [50].

#### Risk Event Alerting

The sharing of sensing output is also an important feature of smart sensing. For example, gas leakage detection in an industrial setting should sound an alarm for the nearby community or the head injury of a user following a collision should be reported to the nearest first-aid point [42,45,47]. Location and position tracking sensors include a GPS, and this output can be transmitted via radio-frequency (RF) transmitters or through the cellular network as a message.

### Common Smart Helmet Sensors

**Figure 6:**
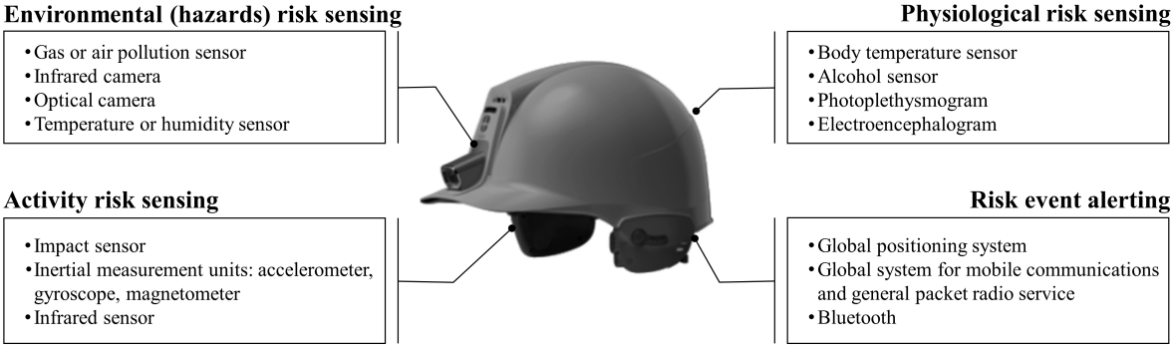
Major risk sensing and adopted sensors.

Typically, a smart helmet consists of a microcontroller to process sensor data; a liquid-crystal display or organic light-emitting diode panel for notification viewing; a light-emitting diode for warning; and a Wi-Fi, RF, Bluetooth, or cellular networking module to transmit data wirelessly. Different sets of sensors can be used to achieve certain functionalities such as detection and reporting of head collisions, air quality checking, or SOS message transmission. Figure 6 presents frequently used sensors in smart helmets. In terms of activity risk sensing, impact sensors and IMU are widely used to detect head injuries [13-16,18,36,37,40,46,52,55,57-61, 63,66,70-73,75-78,80-83], and an IR sensor or force-sensitive resistor (FSR) is used to confirm whether a worker has worn the helmet [36,39,48,49,67-70,73,74,79]. To monitor physiological changes, body temperature, photoplethysmogram, and EEG sensors are adopted [13,37,40,42,43,47,57, 58,64,67,68,72,80,84,86]. Alcohol sensors are used to prevent excessive alcohol use while riding a motorcycle [49,52,69,76-78,82]. To analyze external factors that may put users at risk, gas, temperature, and humidity sensors are used [15-17,36,38,39,42,45,64,67,68]. IR cameras can be used for temperature scanning [18,55,56]. Finally, GPS and Global System for Mobile Communication and General Packet Radio Service sensors are adopted to report nearby accidents [51,55,56,62,65,70,75-78,80,82,83]. Multimedia Appendix 1 [13-18,36-63] summarizes 34 original articles that have reported on the development of prototypes of the proposed smart helmets. Each article is categorized according to the application field, study purpose, the sensor used, wireless protocol, validation method, and assessment score.

### Features of Smart Helmet Sensing

Major features of smart helmets were fall detection, health monitoring, accident prevention, alcohol check, location report, and distress alert as shown in Multimedia Appendix 1. The IMU sensor and accelerometer were adopted to detect sudden changes in head position or acceleration in a certain direction [13-16,18,37,44,46,55,57,59]. For health monitoring, various physiological sensors such as temperature [14,15,39,40,42, 43,47,52,54,57-60], heart rate [14,40,42,43,57,58], humidity sensors [15,38,42,54], and alcohol sensor [49,51,69,76-78,82] were used for continuous monitoring. That is, specific sensors were used to obtain certain features of smart helmets. Although direct readings of the sensor outputs are sufficient for certain features, researchers have also proposed alternatives or inferred other functionalities. For instance, an IR sensor can be used as a proximity sensor as the time for IR reflection depends on the distance between 2 objects. It can also be used to detect whether the helmet was worn or not [36,40,67-70,73]. An FSR is a simple resistor whose resistance changes when an external force is applied, and it can be used to detect if a helmet is being worn by evaluating the resistance changes [48,49,68,74,79]. To check for abnormal head motions, an accelerometer and a gyroscope are often adopted. However, acceleration change or temporal difference in acceleration can also signify abnormal head motions [49]. Furthermore, if the tilt angle measured from the accelerometer remains unchanged for some time, it may be inferred that the user is unconscious [73]. One study used a brushless direct current fan as a velocity sensor as the speed of a fan is proportional to its velocity [48]. Ultrasound sensors and light detection and ranging are often used to measure distance; however, attenuated reflection signals may indicate the density of the material [18] or ground safety [17]. Table 1 summarizes how these features are supported by extracting various modalities from sensors.

**Table 1:** Additional features of smart helmet sensing.

Feature	Type	Sensor	Modality	References
Helmet-wearing check	ARS^a^	IR^b^ sensor	Helmet-to-head proximity or contact	[36,40,67-70,73]
Helmet-wearing check	ARS	Force-sensitive resistor	Resistance to change with a given force	[48,49,68,74,79]
Head motion check	ARS	Accelerometer	3-axis velocity threshold, acceleration variation, the temporal difference in acceleration	[36,40,49,51,52,59,63,66,70,72,75-78,80-82]
Head motion check	ARS	Gyroscope	Angular velocity	[13,61,71,73,83]
Head motion check	ARS	IMU^c^	3-axis inertia change	[55,61]
Driver unconsciousness check	ARS	Accelerometer	Tilt-angle measurement	[73]
Alertness check	PRS^d^	EEG^e^	The ratio of alpha and beta band energy spectrum	[13,37]
Speed check	ERS^f^	Brushless direct current fan	Rotor velocity to voltage	[48]
Floor detection	ERS	LiDAR^g^	Received signal strength indication	[17]
Material detection	ERS	Ultrasound sensor	Signal reflection	[18]

^a^ARS: activity risk sensing.

^b^IR: infrared.

^c^IMU: inertial measurement unit.

^d^PRS: physiological risk sensing.

^e^EEG: electroencephalography.

^f^ERS: environmental risk sensing.

^g^LiDAR: light detection and ranging.

## Discussion

### Principal Findings

In this review, we summarized the published literature on smart helmet technology and the key features of sensors used between 2010 and 2021 (12 years). With the growing demand for smart systems and sensors in the provision of point-of-care, these studies provide possible novel functionalities and propose the potential deployment of smart helmets. Most of these studies have been based in environments in which wearing a helmet is mandatory or suggested, as in occupational health and safety applications, which are widely studied, as shown in Figure 4. Smart helmets can be potentially used in construction [13,17,18,37,39,40,65,66], coal mining [36,38,42,64,68], motorcycle [48-52,69-78,80,82-84], and bicycle riding [54,79], police [56] and firefighting [55,57,58], and health tracking [59-63,85,86] applications. Among the 57 considered articles, there were 4 major domains for smart helmets: ARS, PRS, ERS, and REA, as shown in Figure 5. In addition, the sensors frequently adopted in smart helmets for various purposes were presented in Figure 6. Finally, 34 original articles with proposed prototypes were outlined in Multimedia Appendix 1 and Table 1 according to their key features and validation schemes. In addition, we performed assessment scoring on reviewed articles to show a general tendency of previously done smart helmet research. This paradigm has been adopted in recent artificial intelligence review studies where nonrandomized studies of the effects of the intervention can be potentially biased. In general, the raw cutoff is set to eliminate potentially biased studies, but we skipped this step because of the small number of studies (n=34). The results of assessment scoring revealed that most studies tried to show sensor calibration results or simulations to provide proof of concept. The mean scores of assessment scoring on 5 criteria were 2 out of 3 on the test environment, 2.1 on prototype quality, 1.6 on the feasibility test, 2.3 on sensor calibration, and 2.1 on versatility. Thus, studies of smart helmets lacked feasibility tests for real field use where demonstrations of smart helmets were conducted in controlled laboratory settings. However, worker safety is not only closely related to personal life, but also it can lead to serious accidents such as fire or explosion; for example, a person managing a nuclear power plant is injured. In this concern, smart helmets seem much in demand, but there are a limited number of commercial products in the pilot stage. Therefore, we would like to further discuss further design considerations for personal safety, the general acceptance for deployment in the fields, and potential applications.

### Modular Smart Helmet Design

The main purpose of helmets for military, industrial, and sports use is to protect the brain from external impact. Therefore, smart helmet products need to be tested on various aspects like shock absorption, penetration resistance, eyesight, strap strength, flammability, electrical insulation, and lateral rigidity. The detailed physical and performance requirements can be found on global standards such as ISO 3873:1977 Industrial safety helmets [89], EN 960:2006 Headforms for use in the testing of protective helmets [90], Snell testing [91], and CE (Conformitè Europëenne) product certification. Thus, in the case of smart helmets that are researched and sold, a sensor or module attached to a helmet should meet these international standards. However, designing a modular structure that can be easily detachable may not degrade physical performance and be able to provide smart features. Some representative cases are as follows; LifeBand from SMARTCAP [92] operates with an EEG measurement module that can estimate the condition of workers in real time attached to the helmet strap, and a smart EEG module [93] that can monitor workers’ health status, drowsiness, and poor concentration through an accelerometer, a heart rate sensor, and an EEG sensor from HHS (Health and Happiness System Co. Ltd) is easily attachable on the forehead part inside of standard industrial safety helmets.

The smart sensing module can be configured with various sensor measurements, such as user physiological signals, environmental monitoring, and alerting for location reports. That is, it is possible to design a modular smart helmet that allows users to freely add features which are not available elsewhere, such as improved safety by sensing 360° vision for motorcycle helmets [94] or thermal warning for firefighter helmets [57]. Measurements from modular sensors and linking to backend applications allow direct personalized real-time data recording and interpretation, which enables the creation of applications and services to improve health and health care based on modern IoT paradigms [95,96]. When a modular smart helmet is used in group sensing, different individuals may be equipped with different sets of sensors to improve the quality of environmental sensing with collaborative sensing. Recently, in ECE (Economic Commission for Europe) 22.06 [97], the revised European helmet safety standard that has been in force since January 22, 2022, the crash test, which measures the strength by applying an impact to the helmet from various angles, has been strengthened compared with the previous regulations. Accordingly, the overall helmet shell material could be modified or get thicker which leads to consideration of weight and user comfort.

### Design Considerations

Although the reviewed articles described potential applications and the demand for intelligent systems, they provided little evidence related to the usability and practicality of the proposed device. However, additional metrics such as smart helmet versatility, power consumption, and durability should be determined to examine the usefulness of the system, as well as comfort and ease of use for different population characteristics [98,99]. Without practical applications, user acceptance of smart helmets will not develop [100,101], and consequently, this technology will remain a proof of concept. Furthermore, the weight of smart helmets should be considered to avoid the possibility of neck pain, which has been discussed in the cases of motorcycle helmets and helicopter pilots wearing night glasses [102-104]. If the systems within smart helmets were to become more complicated, such helmets could become more uncomfortable, leading users to be reluctant to wear them. Detailed surveys on usability, such as that in Niforatos et al [53], Zhang [57], and Jeong et al [67], and performance evaluations from daily life trials need to be conducted to ascertain smart helmet usability and reduce the potential reluctance of users by incorporating simple protocols for the number of sensors and user specificity, comfort, including weight, and fashion consideration for the general population.

Communication technologies allow smart helmets to “talk” to each other and to exchange information detected by onboard sensors. To protect and promote health and safety, real-time monitoring of individual data are important in terms of requiring fast responses to incidents and hazards. Connected smart helmets can be implemented with several communication protocols such as Bluetooth, Wi-Fi, Zigbee, and cellular networks. Among reviewed articles, the most widely used protocol was Bluetooth as a representative personal area networking protocol, because a smart helmet does not require wide-area or cellular network communications (eg, LTE and 5G) and can benefit from low energy consumption in the scenarios under consideration [64,80]. The data then can be transmitted to local IoT gateways (eg, dedicated stationary routers or smartphones as mobile routers) to offer data communications. Smartphones could serve as personal mobile gateways that do not have connectivity distance limitations and they are also used to preprocess the data acquired through smart helmets. A low-energy version of Bluetooth or Bluetooth low energy is also suitable for a connectivity feature. When mobile phones are not used as gateways, it is required to install local IoT gateways such as Bluetooth low energy beacons and RF identification in local workplaces which might increase overall management costs [105]. There are extreme work environments where wide-area network communications are not feasible. For example, coal miners lack wide-area network coverage, and thus, existing prototypes usually adopted Zigbee because of the continuously changing working environment and the confined space that causes interferences in communication [106]. A Zigbee unit can be connected to a local area network that supports midrange wireless connectivity and can share information among multiple devices at the same time. Multihop connectivity with Zigbee can enhance data connectivity in extreme environments. In contrast, smart helmets for motorcyclists typically use wide-area network communications, which can be used to send urgent help alerts whenever injuries are detected (eg, sharing location information). Overall, connected helmets assure health and safety by offering continuous risk sensing and real-time incident response. Critical system design requires low-energy usage for long-term use and reliable network connectivity for data exchanges with a suitable network architecture that meets situational user needs.

Another major challenge in the development of smart helmets is personal information collection and privacy infringement. With the advancement of the IoT, real-time monitoring data are shared and analyzed to find factors related to events. Although this monitoring is supposed to assist users, some aspects of personal privacy are violated [107-111]. Prior studies have shown that privacy concerns related to wearable cameras are often influenced by the social, behavioral, and environmental contexts of users [112]. Wearable camera users are often conscious of bystander privacy, and likewise, bystanders are concerned about potential privacy violations (eg, the subtleness and ease of recording) [113]. Advanced data processing may also have privacy implications. However, the current studies utilizing wearable optical cameras for image transmission [55,56], resource management [65], and facial recognition [114] lack privacy considerations. Furthermore, personal physiological data or location information can be misused, possibly associated with poor data management policies. In such scenarios, health monitoring results may encourage the tracking of work performance (ie, using the data for a secondary purpose without explicit consent). This practice may influence the performance review of workers and cause monitoring to become surveillance (beyond health monitoring). Beyond secondary use, the security of the devices themselves can also be problematic as the low computing power within smart helmet systems may make them vulnerable to unauthenticated access [115,116]. As smart helmet technology is still in its infancy, such implications are not yet fully understood and should be considered as part of future research and implementation.

### Emerging Applications of Smart Helmets

A recent example of a promising smart helmet is a helmet with a thermal camera to assist in monitoring the COVID-19 outbreak [14,23,87,88]. This KC N901 can measure the body temperatures of people in a crowd with an accuracy of 0.3 °C using an IR camera, as well as scan the QR codes of individuals, recognize license plates, and recognize people using an optical camera with facial recognition functionality [114]. It can detect a person with a high temperature and transmit the location and identity of that person. According to the manufacturer data, the helmet weighs around 1 kg, can measure the temperatures of 200 people in a minute, and has a battery life of 5 hours in temperature scan mode, thereby showing promise as a mobile monitoring system. However, helmets with thermal imaging also need to be able to determine the causes of increased temperature to avoid false positives and unnecessary intervention or contact tracing, which often occurs in menopausal women, ill individuals, postexercise, and pregnant women. The latter factor is not only important in terms of individual privacy but can also be applied to provide fetal health indicators [117]. Another emerging application of smart helmets is related to electric scooters. Since the introduction of urban rental programs, major injuries associated with electric scooters have included head injuries, as users rarely wear helmets while riding scooters [118,119]. This recently introduced mode of transportation continues to expand because of its usability and low cost, but there have been little to no efforts to establish safety regulations. Mitchell et al [120] proposed a correlation between wearing a helmet and decreased risk of head injury in cases of alcohol consumption. Smart helmets may help encourage users to wear helmets by incorporating electric scooter ignition-lock systems with helmet-wearing checks [48,49,69,70,73,74,79], alcohol checks [49,52,69,76-78,82], and SOS signal sending capabilities [51,70,75-78,80,82,83].

### Conclusions

This paper comprehensively reviewed the recent trends in smart helmet technology. The primary uses of smart helmets for health and safety were explored, and the most relevant applications were described, as well as the sensors adopted to enable key features. Furthermore, the most relevant examples of smart helmet applications were detailed, showing their potential uses. The current focus on smart helmets are industrial safety helmets and motorcyclist helmets, and there are growing application fields for first responders and general health tracking where health and safety matter. Smart helmets play key roles in sensing capabilities, actuations, and distress alerts. Finally, the main barriers, challenges, and recommendations for the deployment of smart helmets were discussed. In summary, this paper presents the current status of smart helmet technology, main issues, and prospects for future smart helmet designers and developers with the objective of making the smart helmet concept a reality.

## Appendix

Multimedia Appendix 1 Summary of original articles.

## Abbreviations

ARS: activity risk sensing
ECG: electrocardiogram
EEG: electroencephalogram
ERS: environmental risk sensing
FSR: force sensing resistor
IMU: inertial measurement units
IoT: Internet of Things
IR: infrared
ISH: industrial safety helmet
PRISMA: Preferred Reporting Items for Systematic Reviews and Meta-Analyses
PRS: physiological risk sensing
REA: risk event alerting
RF: radio frequency
