# Supplementary material for: Trends in Smart Helmets With Multimodal Sensing for Health and Safety: Scoping Review
Source: JMIR Mhealth Uhealth. 2022 Nov 15;10(11):e40797. doi: 10.2196/40797 (PMC9709670; doi:10.2196/40797)
Supplement: Multimedia Appendix 1 [file mhealth_v10i11e40797_app1.docx]

**Trends in Smart helmets With Multimodal Sensing for Health and Safety: Scoping Review.**

**Multimedia Appendix 1**

[Lee P](https://www.ncbi.nlm.nih.gov/pubmed/management/validator/811C713D00AF/citations/?start=0)^1^, [Kim H](https://www.ncbi.nlm.nih.gov/pubmed/management/validator/811C713D00AF/citations/?start=0)^1^, [Zitouni MS](https://www.ncbi.nlm.nih.gov/pubmed/management/validator/811C713D00AF/citations/?start=0)^2^, [Khandoker A](https://www.ncbi.nlm.nih.gov/pubmed/management/validator/811C713D00AF/citations/?start=0)^3,4^, [Jelinek HF](https://www.ncbi.nlm.nih.gov/pubmed/management/validator/811C713D00AF/citations/?start=0)^3,4^, [Hadjileontiadis L](https://www.ncbi.nlm.nih.gov/pubmed/management/validator/811C713D00AF/citations/?start=0)^3,4,5^, [Lee U](https://www.ncbi.nlm.nih.gov/pubmed/management/validator/811C713D00AF/citations/?start=0)^6,1^, [Jeong Y](https://www.ncbi.nlm.nih.gov/pubmed/management/validator/811C713D00AF/citations/?start=0)^7,1^.

[**Author information**](https://www.ncbi.nlm.nih.gov/pubmed/management/validator/811C713D00AF/citations/?start=0#afflist1)

**1** KAIST Institute for Health Science and Technology, Korea Advanced Institute of Science and Technology, Daejeon, KR.

**2** College of Engineering and IT, University of Dubai, Dubai, AE.

**3** Department of Biomedical Engineering, Khalifa University of Science and Technology, Abu Dhabi, AE.

**4** Healthcare Engineering Innovation Center, Khalifa University of Science and Technology, Abu Dhabi, AE.

**5** Department of Electrical and Computer Engineering, Aristotle University of Thessaloniki, Thessaloniki, GR.

**6** School of Computing, Korea Advanced Institute of Science and Technology, Daejeon, KR.

**7** Department of Bio and Brain Engineering, Korea Advanced Institute of Science and Technology, 291 Daehak-ro Yuseong gu, Daejeon, KR.

Corresponding Author:

Yong Jeong

Department of Bio and Brain Engineering,

Korea Advanced Institute of Science and Technology,

Daejeon, 34141, South Korea

Tel.: +82-42-350-4324

Email: yong@kaist.ac.kr

**Table S1.** Summary of original articles.

| Field | | Study | Study purpose | Sensor used | | Application | Protocol | Validation | Assessment score |
| --- | --- | --- | --- | --- | --- | --- | --- | --- | --- |
| Industry | | Seo et al [17] | ARS^a^: collision sensing; ERS^b^: air quality, floor safety sensing | Ultrasonic sensor, CO^c^ sensor, and 3-axis accelerometer | | Accident prevention | Bluetooth | Algorithm test: floor safety detection result | - 13/15 - A/A/B/A/B^d^ |
| Industry | | Behr et al [36] | ARS: collision sensing, helmet-wearing check ERS: air quality sensing | Gas sensor, vibration sensor, IR^e^ sensor | | Helmet-wearing check | Zigbee | Sensor test: gas sensor test, IR sensor test, impact test, wireless transmission test | - 7/15 - C/C/C/B/B |
| Industry | | Li et al [37] | ARS and PRS^f^: recognize anomalous behavior | IMU^g^ sensor, EEG^h^ | | Health monitoring; accident prevention | Bluetooth | Algorithm test: risk level determination | - 11/15 - B/B/B/B/A |
| Industry | | Pirkl et al [18] | ERS: material detection, self-localization, room dimension estimation, temperature scan | LiDAR^i^, IMU, ultrasound sensor, IR camera | | User enhancement | None, head-mounted display | Algorithm and sensor test: position estimation, room dimension estimation, ultrasound material detection, IR camera scan | - 14/15 - A/A/B/A/A |
| Industry | | Dhole et al [13] | ARS: fall detection PRS: detect drowsiness | IMU sensor; EEG | | Fall detection; accident prevention | Wi-Fi | Algorithm test: activity classification result, activity plot | - 13/15 - B/A/A/A/B |
| Industry | | Harshitha et al [38] | ERS: air quality, temperature, and humidity sensing | Gas sensor, temperature sensor, humidity sensor | | Accident prevention | Zigbee | Sensor test: sensor readings transmission | - 5/15 - C/C/C/C/C |
| Industry | | Altamura et al [39] | ARS: helmet-wearing check; PRS: heart beat; ERS: air quality check | Gas sensor, temperature sensor, pulse sensor | | Helmet-wearing check | Zigbee | Sensor test: transmission test | - 11/15 - B/B/B/A/B |
| Industry | | Mehata et al [40] | ARS: fall detection; PRS: vital monitoring | Temperature sensor, heart beat sensor, IR sensor, accelerometer | | Fall detection; health monitoring | Wi-Fi | Sensor test: heart beat measure, fall detection | - 7/15 - C/C/B/B/C |
| Industry | | Kim et al [41] | ARS: proximity warning | Bluetooth receiver | | Accident prevention | Bluetooth | Sensor test: signal transmission strength by distance | - 11/15 - A/A/C/A/C |
| Industry | | Vishnukumar et al [42] | PRS: heart rate sensing; ERS: working environment risk sensing; REA^j^: accident alert | Heart beat sensor, humidity sensor, gas sensor, temperature sensor | | Health monitoring; Accident prevention | Zigbee | Sensor test: sensor readings | - 8/15 - C/B/C/B/B |
| Industry | | Hinge et al [14] | ARS: fall detection; PRS: vital monitoring | Heart beat sensor, temperature sensor, IMU sensor | | Fall detection; Health monitoring | Bluetooth | Algorithm test: risk level determination | - 6/15 - C/C/C/B/C |
| Industry | | Sharma et al [43] | ARS: heart rate, skin temperature sensing; ERS: temperature sensing | Heart rate sensor, temperature sensor | | Health monitoring | Bluetooth | Sensor test: heat stress indexes, physiological and environmental variables | - 13/15 - A/B/A/A/B |
| Industry | | Kim et al [44] | ARS: helmet-wearing check | IMU sensor | | Helmet-wearing check | Bluetooth | Algorithm test: helmet-wearing check while in action | - 13/15 - B/B/A/A/A |
| Industry | | Colombo et al [45] | ERS: gas and noise sensing; REA: distress alert | Gas sensor, noise sensor | | Accident prevention; accident alert | Wi-Fi | User assessment survey | - 11/15 - A/A/C/C/A |
| Industry | | Marquez et al [15] | ARS: detect external impact; ERS: air quality check, luminosity sensing | FSR^k^, IMU, gas sensor, humidity sensor, temperature sensor, luminosity sensor | | Accident prevention | Wi-Fi | Algorithm and sensor test: risk identification and sensor readings | - 13/15 - A/B/A/A/B |
| Industry | | Ngubo et al [46] | ARS: sensing worker mobility, helmet and shoe wearing check, and load weighting | IMU, weight sensor, light sensor | | Accident prevention | Zigbee | Sensor test: weight sensor readings and helmet-wearing check | - 7/15 - C/B/C/B/C |
| Industry | | Lee et al [16] | ARS: fall detection; ERS: gas leakage and object falling sensing | IMU, gas sensor, ultrasonic sensor | | Fall detection; accident prevention | Bluetooth | Sensor test: sensor readings | - 9/15 - B/B/C/B/B |
| Industry | | Angelia et al [47] | ARS: monitor pulse rate, temperature, location;  REA: distress alert | Pulse rate sensor, temperature sensor, impact sensor, GPS | | Health monitoring; accident alert | RF^l^ (sub-GHz ISM^m^ bands) | Algorithm and sensor test: pulse rate and temperature readings, distress signal testing | - 11/15 - B/B/A/B/B |
| Sports | | Rasli et al [48] | ARS: speed check, collision detection, helmet-wearing check | FSR, BLDC^n^ fan | | Helmet-wearing check | Bluetooth | Sensor test: sensor readings | - 7/15 - B/C/C/B/C |
| Sports | | Tapadar et al [49] | ARS: helmet-wearing check, accident detection; PM: alcohol check | | Flex sensor, impact sensor, accelerometer, breath-analyzer | Helmet-wearing check; alcohol check | None | Algorithm and sensor test: helmet wear detection test, accident report test, alcohol test | - 10/15 - B/C/A/B/B |
| Sports | Rupanagudi et al [50] | | ERS: rear vehicle intimation and collision avoidance | | Camera | Accident prevention | GSM^o^ | Algorithm test: performance tested | - 13/15 - A/A/B/A/B |
| Sports | Sasurekha et al [51] | | AM: collision detection; REA: accident reporting | | Impact sensor, accelerometer, GSM or GPS | Accident alert | Bluetooth | Algorithm and sensor test:  Impact sensor, accelerometer test, SMS simulation | - 7/15 - B/C/C/B/C |
| Sports | Mangno et al [52] | | ARS: fall detection; PRS: alcohol control | | Accelerometer, temperature sensor, alcohol detector | Fall detection; alcohol check | None | Sensor test: current consumption, battery life test | - 8/15 - C/C/C/B/A |
| Sports | Niforatos et al [53] | | ERS: rear skier intimation and collision avoidance | | LiDAR | Accident prevention | Bluetooth | User assessment survey | - 7/15 - C/A/C/C/C |
| Sports | Youssef et al [54] | | PRS: measure helmet thermal comfort | | Humidity, temperature sensor, PPG^p^ sensor | Health monitoring | Wi-Fi | Sensor test: indoor test | - 12/15 - B/A/C/A/A |
| First responder | Jeong et al [55] | | ARS: fall detection; PRS: oxygen residual check; ERS: temperature scan | | IR, optical camera, oxygen residual sensor, IMU, GPS | Fall detection; Health monitoring | GSM | Sensor test: fall detection test, temperature scan test | - 11/15 - B/A/C/B/A |
| First responder | Mohammed et al [56] | | ERS: temperature scan, localization | | IR camera, optical camera, GPS | User enhancement | Wi-Fi | Algorithm and sensor test: location history system, thermal image demonstration test | - 6/15 - C/C/C/C/B |
| First responder | Zhang et al [57] | | ARS: fall detection; PRS: heart rate and temperature sensing; REA: alert potential dehydration | | Heart rate sensor, temperature sensor, IMU | Fall detection; health monitoring | Bluetooth | User assessment survey | - 11/15 - A/A/C/C/A |
| First responder | Choi et al [58] | | ARS: acceleration; PRS: temperature, heart rate | | Temperature sensor, heart rate sensor, accelerometer | Health monitoring | Wi-Fi | Algorithm and sensor test: distance testing from acceleration data | - 12/15 - A/A/C/A/B |
| Health tracking | Rosenberg et al [59] | | ARS: fall detection; PRS: temperature scan, oxygen residual check | | EEG, ECG^q^, and IMU | Fall detection; health monitoring | Bluetooth | Sensor test: EEG and ECG test | - 13/15 - B/A/B/A/A |
| Health tracking | Strangman et al [60] | | PRS: multimodal monitoring | | EEG, ECG, NIRS^r^, accelerometer, gyroscope, respiration, temperature sensor | Health monitoring | Bluetooth | Algorithm and sensor test:  Outdoor test result, head motion detection | - 11/15 - B/B/C/A/A |
| Health tracking | Shahiduzzaman et al [61] | | ARS: fall detection | | Camera, accelerometer, gyroscope | Fall detection | None | Algorithm test: performance tested | - 8/15 - B/C/B/B/C |
| Health tracking | Tang and Li [62] | | ARS: all time indoor monitoring of older adults | | Doppler radar, gyroscope | Location tracking | Radar | Algorithm and sensor test:  Outdoor test result, brain motion detection | - 12/15 - A/A/B/A/C |
| Health tracking | Lim et al [63] | | PRS: real-time health monitoring | | EEG, ECG, oxygen saturation, accelerometer | Health monitoring | LoRa^s^ | Algorithm and sensor test: blast source localization simulation | - 8/15 - C/C/C/B/A |

^a^ARS: activity risk sensing.

^b^ERS: environmental risk sensing.

^c^CO: carbon monoxide.

^d^A: attribute score 3; B: attribute score 2; C: attribute score 1.

^e^IR: infrared.

^f^PRS: physiological risk sensing.

^g^IMU: inertial measurement unit.

^h^EEG: electroencephalography.

^i^LiDAR: light detection and ranging.

^j^REA: risk event alerting.

^k^FSR: force-sensitive resistor.

^l^RF: radio frequency.

^m^ISM: Industrial, Scientific and Medical

^n^BLDC: Brushless Direct Current

^o^GSM: Global System for Mobile Communications.

^p^PPG: photoplethysmogram.

^q^ECG: electrocardiogram.

^r^NIRS: near-infrared spectroscopy

^s^LoRa: long range radio.
